# Transcriptomic analysis reveals pathophysiological relationship between chronic obstructive pulmonary disease (COPD) and periodontitis

**DOI:** 10.1186/s12920-022-01278-w

**Published:** 2022-06-08

**Authors:** Shuqin Liu, Yun Fu, Dirk Ziebolz, Simin Li, Gerhard Schmalz, Fan Li

**Affiliations:** 1 Department of Stomatology, Jiangxi Provincial People’s Hospital, The First Affiliated Hospital of Nanchang Medical College, Aiguo Road No. 152, Nanchang, 330006 Jiangxi Province China; 2 Department of General Practice, Jiangxi Provincial People’s Hospital, The First Affiliated Hospital of Nanchang Medical College, Aiguo Road No. 152, Nanchang, 330006 Jiangxi Province China; 3 Department of Cariology, Endodontology and Periodontology, University Leipzig, Liebigstr. 12, 04103 Leipzig, Germany; 4 Stomatological Hospital, Southern Medical University, Guangzhou, 510280 Guangdong Province China; 5 Department of Pulmonary and Critical Care Medicine, Jiangxi Provincial People’s Hospital, The First Affiliated Hospital of Nanchang Medical College, Aiguo Road No. 152, Nanchang, 330006 Jiangxi Province China

**Keywords:** COPD, Periodontitis, Inflammation, Bioinformatics

## Abstract

**Background:**

The aim of this study was to detect potential crosstalk genes, pathways and immune cells between periodontitis and chronic obstructive pulmonary disease (COPD).

**Methods:**

Chronic periodontitis (CP, GSE156993) and COPD (GSE42057, GSE94916) datasets were downloaded. Differential expressed genes (DEGs; *p* < 0.05) were assessed and screened for overlapping results, following functional pathway enrichment analyses (*p* < 0.05). The xCell method was used to assess immune cell infiltration relationship between CP and COPD. Features of the detected cross-talk genes were revealed using conventional Recursive Feature Elimination (RFE) algorithm in R project. Receiver-operating characteristic curves were applied to evaluate the predictive value of the genes. Furthermore, Pearson correlation analysis was performed on crosstalk markers and infiltrating immune cells in CP and COPD, respectively.

**Results:**

A total of 904 DEGs of COPD and 763 DEGs of CP were acquired, showing 22 overlapping DEGs between the two diseases. Thereby 825 nodes and 923 edges were found in the related protein–protein-interaction network. Eight immune cell pairs were found to be highly correlated to both CP and COPD (|correlation coefficients |> 0.5 and *p*-value < 0.05). Most immune cells were differently expressed between COPD and CP. RFE identified three crosstalk genes, i.e. EPB41L4A-AS1, INSR and R3HDM1. In correlation analysis, INSR was positively correlated with Hepatocytes in CP (r = 0.6714, *p* = 0.01679) and COPD (r = 0.5209, *p* < 0.001). R3HDM was positively correlated with Th1 cells in CP (r = 0.6783, *p* = 0.0153) and COPD (r = 0.4120, *p* < 0.01).

**Conclusion:**

EPB41L4A-AS1, INSR and R3HDM1 are potential crosstalk genes between COPD and periodontitis. R3HDM was positively correlated with Th1 cells in both diseases, while INSR was positively correlated with Hepatocytes in periodontitis and COPD, supporting a potential pathophysiological relationship between periodontitis and COPD.

## Introduction

Periodontitis is an opportunistic, multifactorial inflammatory disease, affecting the periodontal tissues, i.e., the marginal gingiva, periodontal ligament and alveolar bone [1]. During the disease process, progressive destruction of both soft and hard tissue occurs, finally resulting in tooth loosening and loss in the end-stage of the disease [1]. However, these inflammatory processes and potential consequences are not only restricted to the oral cavity; different effects of oral, especially periodontal diseases on systemic health are known [2]. Especially, relationships between periodontitis and non-communicable diseases, including diabetes or cardiovascular diseases are evident [2].

In this context, periodontitis is also potentially related to respiratory diseases, including asthma, pneumonia and chronic obstructive pulmonary diseases (COPD) [3–5]. COPD is a highly prevalent disease, causing over 3 million deaths worldwide each year; thereby, it is a chronic pulmonary disease with different complex underlying pathophysiological mechanisms [6]. A recent systematic review and meta-analysis revealed a relationship between periodontitis and COPD, whereby COPD patients had a 1.78-fold increased risk of having periodontitis [3]. Generally, both diseases share risk factors, whereby cigarette smoking is related to both, periodontitis and COPD [7, 8]. Furthermore, diabetes mellitus, obesity and the metabolic syndrome are potentially related with these two diseases [9, 10]. Thereby, obesity directly influences lung function [11], while diabetes mellitus and the metabolic syndrome are common systemic manifestations of COPD [9]. A neutrophil-related inflammation and related increased activity of the immune response underline the similar pathophysiology between COPD and periodontal diseases [5]. The shared pathophysiology between periodontitis and COPD might rely on an amplification of neutrophilic inflammation and altered neutrophil functions [12]. Although the recent literature supports a shared pathophysiology, it is still questionable, whether the relationship between periodontitis and COPD would be causal or a coincidental occurrence of these two diseases [3]. A potential causality might be supported by the hypothesis of COPD as a chronic systemic inflammatory syndrome, whereby COPD is not a disease restricted to the airways, but a complex chronic inflammatory condition [13]. Thereby, autoimmunity might play an important role, which is relevant for disease progression in both, periodontitis and COPD [14, 15]. Furthermore, especially the role of neutrophils as a key effector cell in inflammation is supposed to be involved in the causal interrelationship between periodontitis and COPD [5]. Accordingly, a shared or at least similar pathophysiology between these two diseases appears conceivable; however, more research in the field is still needed to gain a deeper understanding of the potential interrelationships and biological processes [3].

Bioinformatics analysis recently was able to reveal several potential cross-talk genes and related pathways between oral and systemic diseases, e.g. periodontitis and Alzheimer´s disease or Rheumatoid arthritis [16, 17]. Therefore, this current study used bioinformatics to examine the relationship between periodontitis and COPD. It was aimed to examine potential crosstalk genes, which are shared between the two diseases. Moreover, the interaction between those potential crosstalk genes and infiltrating immune cells should be assessed to gain a deeper insight into the pathophysiological processes, which may link periodontitis and COPD. Altogether, the main objective was to analyze the shared genetic mechanisms between COPD and periodontitis and their relation to immune cell infiltration. Therefore, publicly available data should be assessed and examined regarding a genetic overlap between the two diseases. Furthermore, infiltrating immune cells, which were regulated by or related to the respective crosstalk genes, should be detected and analyzed regarding their correlation with the identified crosstalk genes. It was hypothesized that different crosstalk genes exist between periodontitis and COPD, which are related to infiltrating immune cells, mediating the pathophysiological interrelation between both diseases.

## Materials and methods

This study was designed as a bioinformatics study and based on publicly available datasets. The analytic procedure is described in the following.

### Data download

**Fig. 1:**
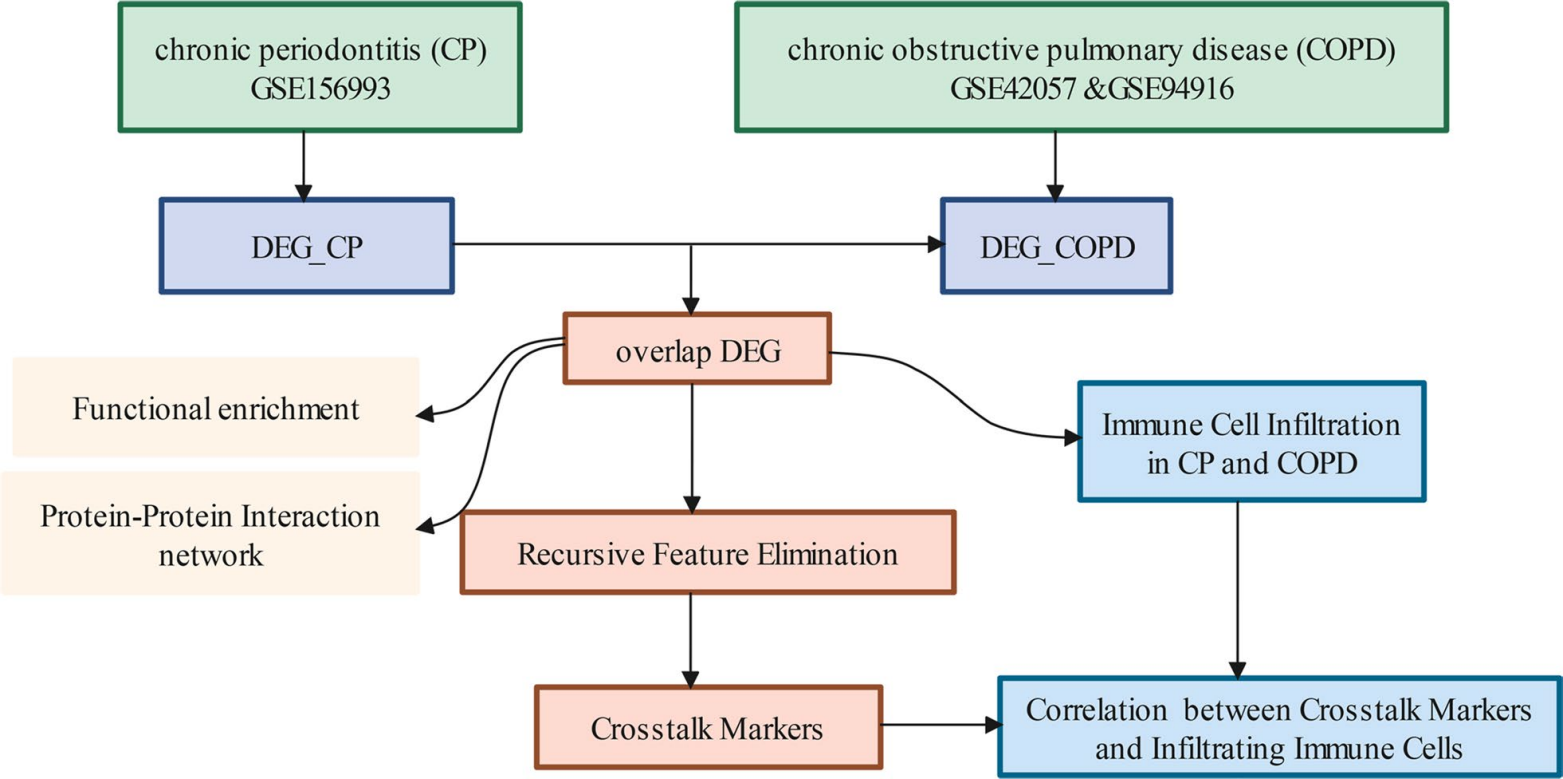
The framework of the current study

The workflow of data analysis is shown in Fig. 1. First, gene expression of chronic periodontitis (CP) and chronic obstructive pulmonary disease (COPD) was downloaded from the GEO (https://www.ncbi.nlm.nih.gov/geo/) database [18]. For CP and COPD, the detailed information of samples was displayed in Table 1.

**Table 1:** The data summary of chronic periodontitis (CP) and chronic obstructive pulmonary disease (COPD)

Disease	Dataset	Platform	Case samples	Control samples
COPD	GSE42057	GPL570	94	42
	GSE94916	GPL20844	6	6
CP	GSE156993	GPL570	6	6

### Data preprocessing

For the expression data, the probe ID to gene symbol was mapped and the gene expression level for the same gene was normalized with the average gene expression. To analyze COPD, the GSE42057 and GSE94916 gene expression matrices based on the common genes were combined, and the inter-batch difference was removed using the ComBat method of “sva” package of R project [19]. ComBat function allows users to adjust for batch effects in datasets where the batch covariate is known by using methodology described in Johnson et al. [20]. It uses either parametric or non-parametric empirical Bayes frameworks for adjusting data for batch effects. Users receive an expression matrix that has been corrected for batch effects. The input data are assumed to be cleaned and normalized before batch effect removal. Moreover, the two-dimensional PCA cluster was applied to check whether the batch difference was removed.

### Differentially expressed genes (DEGs) analysis

The “limma” package of R project [21] was applied to analyze the differentially expressed genes for CP and COPD merged data, respectively. The genes with *p* < 0.05 were considered as statistically significant. Among these significantly differentially expressed genes, those genes with log2FC > 0 were up-regulated and genes with log2FC < 0 were down-regulated, because the value of log2FC in the analyzed data was low. The overlapping genes between CP and COPD were interpreted as the potential crosstalk genes.

### Functional enrichment analysis

The “clusterProfiler” package of R project [22] was used to perform the Gene Ontology (GO) biological process and KEGG pathway enrichment analyses for the potential crosstalk genes. The functions with *p* < 0.05 were considered significant enrichment.

### Construction of protein–protein interaction network

The protein–protein interactions of the 22 potential crosstalk genes were downloaded from BIOGRID (Biological General Repository for Interaction Datasets) [23], HPRD (Human Protein Reference Database) [24], DIP (Database of Interacting Proteins) [25], MINT (Molecular INTeraction database) [26], PINA (Protein Interaction Network Analysis) [27], InnateDB (A knowledge resource for innate immunity interactions & pathways) [28] and Instruct (3D protein interactome networks with structural resolution) [29]. The Cytoscape platform [30] was used to construct the protein–protein interaction network and for analysis of the network topological characteristics.

### Immune cell infiltration analysis

The gene expression of potential crosstalk genes was assessed in the CP (GSE156993) and COPD (merged data of GSE42057 and GSE94916) samples. Subsequently, the immune cell infiltration relationship between CP and COPD in the crosstalk process was evaluated by using the xCell method (https://github.com/dviraran/xCell) [31]. xCell is a gene signature-based method learned from thousands of pure cell types from various sources, which include 64 immune and stromal cell types. To analyze the differentially expressed cells between CP and COPD, the cell enrich score of immune cells in the disease samples was extracted and the difference was analyzed using Wilcoxon test.

### Identification of crosstalk markers

The expression values of the potential crosstalk genes were assessed from the CP and the merged COPD data, and the feature selection was performed by the conventional Recursive Feature Elimination (RFE) algorithm in R project [32]. The screened feature genes were interpreted as crosstalk markers. Furthermore, the gene expression value of the crosstalk markers was extracted from the CP and the merged COPD gene expression profile, and then the prediction was assessed by receiver-operating characteristic (ROC) curves with the pROC package [33] and displayed using the ggplot2 package in R [34].

### Correlation analysis between crosstalk markers and infiltrating immune cells

A Pearson correlation analysis of crosstalk markers and infiltrating immune cells in CP and COPD was applied, respectively. The enrichment score of each type of immune cells in the samples was obtained by performing xCell analysis [31]. The enrichment scores in any two types of cells were obtained and used for the Pearson correlation coefficient analysis [35]. The Pearson correlation coefficient (r) between any two types of immune cells was calculated, which ranged between − 1 and 1. If the r-value is greater than zero, the correlation between two cells was positive. This positive correlation means that, when the enrichment score of cell_A becomes larger, then its positively correlated cell_B’ enrichment score will also become larger. An r-value of less than zero indicates negative correlation, which means that, when the enrichment score of cell_A becomes larger, then its negatively correlated cell_B’s enrichment score becomes smaller. The greater the absolute value of r, the more significant the correlation will be. When |r| is close to 1, it indicates a perfect correlation; and when |r| is close to 0, it indicates no correlation. Generally, a value of |r| greater than 0.9 is considered as an excellent correlation; a value of |r| greater than 0.7 is considered a strong correlation; a value of |r| between 0.5 and 0.7 is a moderate correlation, and a value of |r| less than 0.4 is considered a weak or no correlation. After performing the correlation analysis, the “ggplot2” package [34] was used to visualize the results.

## Results

### Data preprocessing

**Fig. 2:**
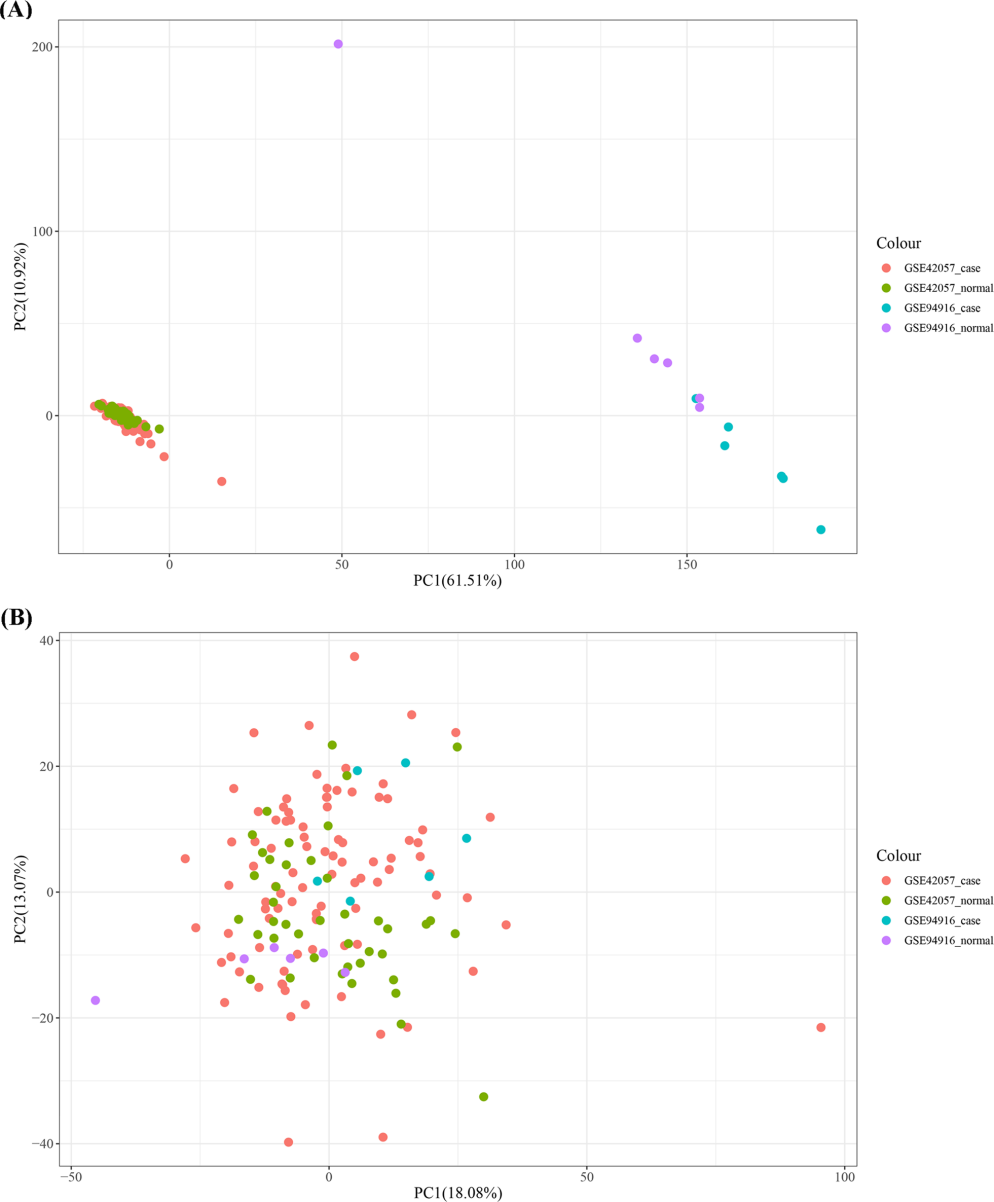
Principal component analysis (PCA) cluster plot before (**A**) and after (**B**) sample merge between GSE42057 and GSE94916

Before normalization, COPD datasets GSE42057 and GSE94916 were obviously different and thus divided into two parts (Fig. 2A). After normalization, the samples of GSE42057 and GSE94916 were merged together and the differences between the two samples had been significantly reduced and thus were reliable for further analysis (Fig. 2B).

### Differentially expressed gene analysis

**Fig. 3:**
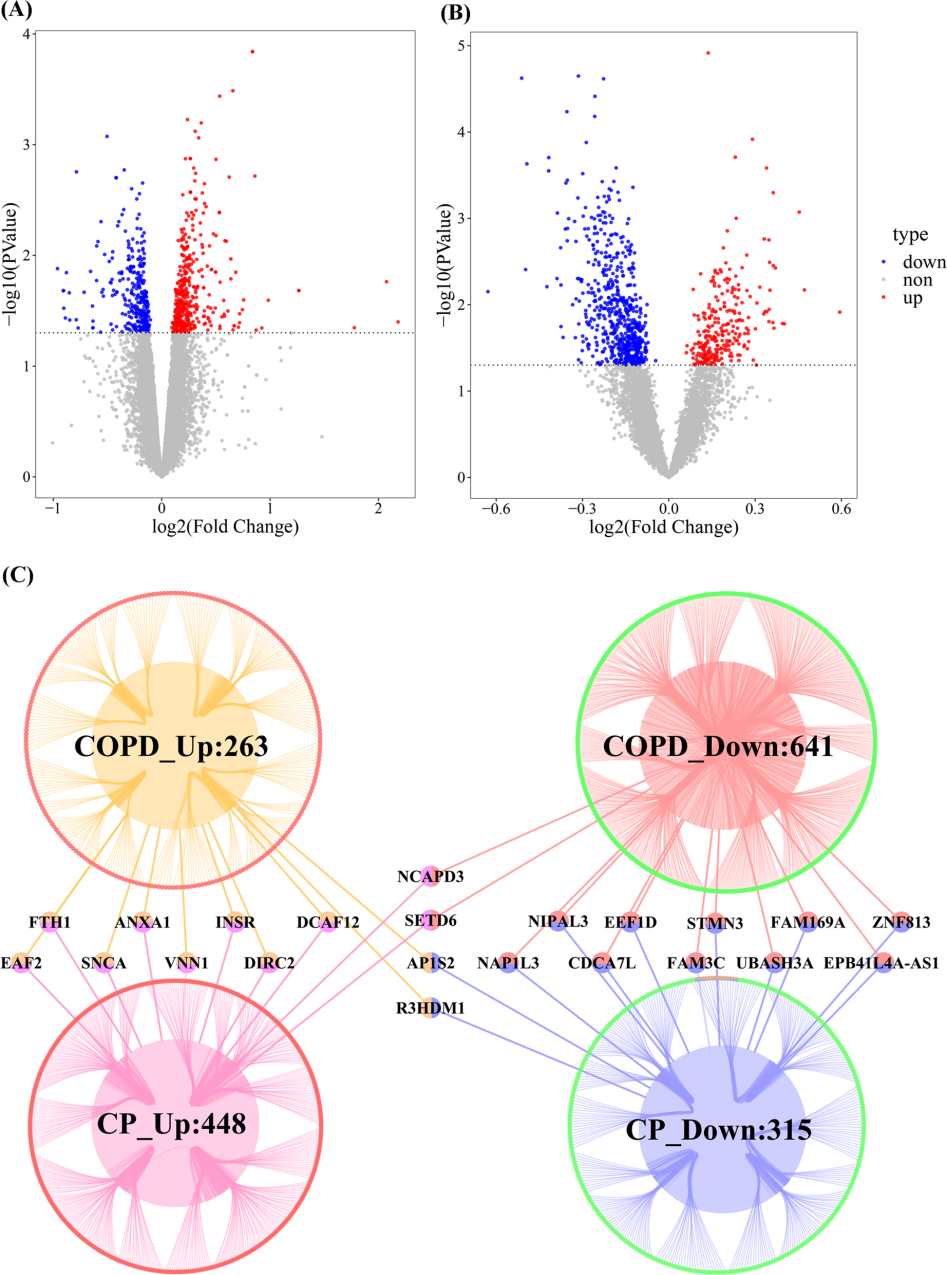
(**A**) and (**B**) show the Volcano map of deregulated genes (DEGs) for periodontitis (CP) and chronic obstructive pulmonary disease (COPD), respectively. Red represents up-regulated differentially expressed genes, grey represents not significantly different genes, and blue represents down-regulated differentially expressed genes. **C** The overlapped DEGs between CP and COPD

Figure 3A, B show the gene expression of CP and merged COPD data. Finally, 904 DEGs of COPD and 763 DEGs of CP were acquired (Table 2). Thereby, 22 overlapping genes between CP and COPD were found, which were the potential crosstalk genes between CP and COPD (Fig. 3C).

**Table 2:** DEG number of COPD and CP

	COPD	CP
DEG_up	263	448
DEG_down	641	315
Total	904	763

### Function enrichment and PPI network for the potential crosstalk genes

**Fig. 4:**
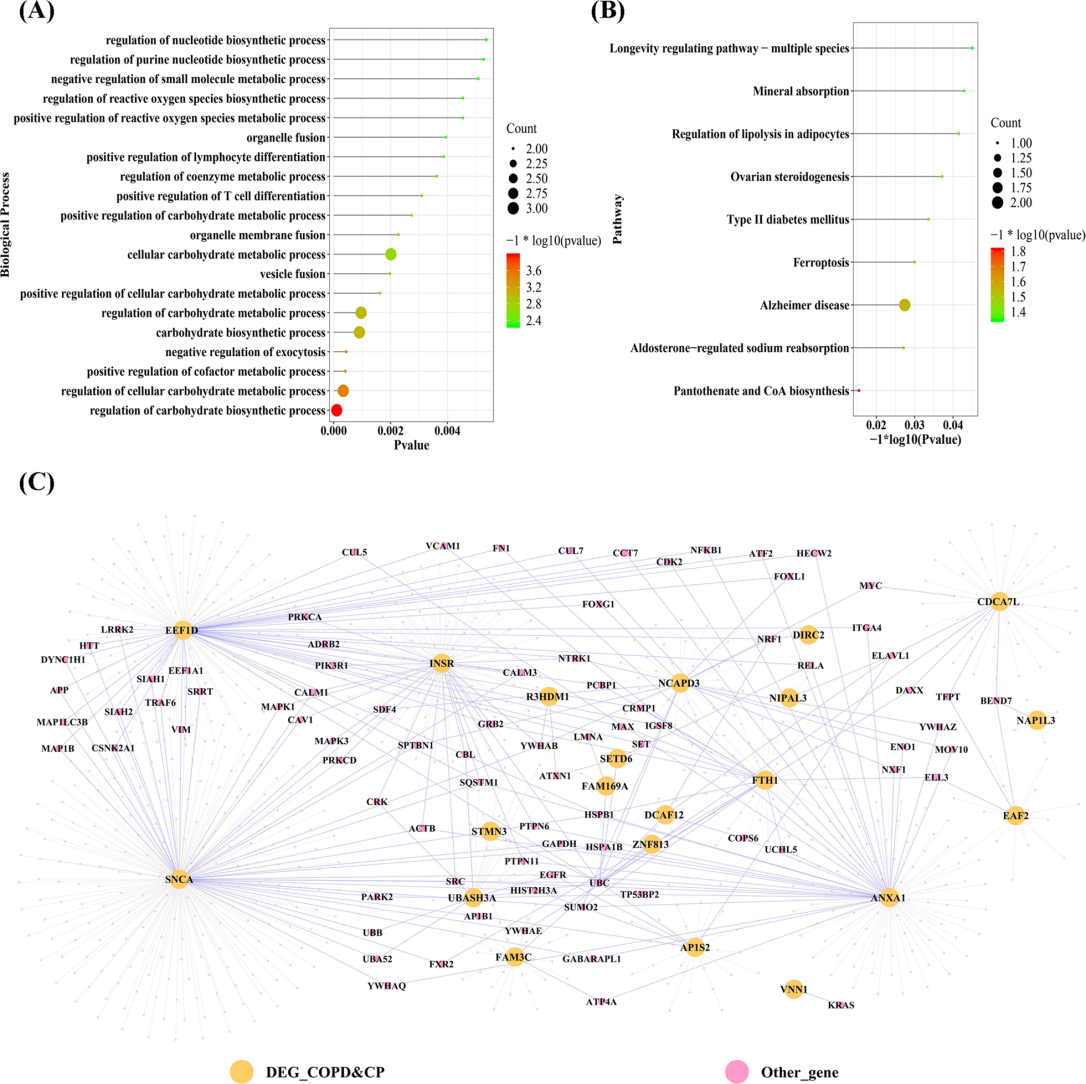
The significant enriched biological processes (**A**) and pathways **B** of 22 potential crosstalk genes. **C** The protein–protein interaction network for 22 potential crosstalk genes. In the network, the size of node indicated the higher degree of node

With the clusterProfiler of R project [22], several biological processes for the 22 potential crosstalk genes could be revealed (Fig. 4A). Besides, the potential crosstalk genes regulated nine biological pathways, including the Pantothenate and CoA biosynthesis, Aldosterone-regulated sodium reabsorption, Ferroptosis, Regulation of lipolysis in adipocytes as well as type II diabetes mellitus (Fig. 4B). For the 22 potential crosstalk genes, a PPI network was constructed, which included 825 nodes and 923 edges (Fig. 4C). The network topological characteristics of PPI network were analyzed, and the top 20 nodes were extracted, which are displayed in Table 3.

**Table 3:** the topological characteristic of top 20 nodes in the PPI network

Gene	Label	Degree	Average shortest path length	Betweenness centrality	Closeness centrality	Topological coefficient
SNCA	COPD&CP	249	2.424574	0.476994	0.412444	0.017938
EEF1D	COPD&CP	160	2.645985	0.303017	0.377931	0.027917
ANXA 1	COPD&CP	117	2.753041	0.222923	0.363235	0.034188
INSR	COPD&CP	114	2.753041	0.22763	0.363235	0.025146
FTH1	COPD&CP	56	2.867397	0.126682	0.348748	0.038095
NCAP D3	COPD&CP	47	2.913625	0.088015	0.343215	0.045213
CDCA 7L	COPD&CP	45	2.877129	0.122311	0.347569	0.028758
AP1S2	COPD&CP	31	2.957421	0.065846	0.338132	0.047312
FAM3 C	COPD&CP	28	2.969586	0.062619	0.336747	0.038265
UBAS H3A	COPD&CP	24	2.979319	0.041695	0.335647	0.068452
EAF2	COPD&CP	16	4.658151	0.031841	0.214677	0.083333
UBC		15	2.036496	0.322608	0.491039	0.074936
DCAF 12	COPD&CP	10	3.006083	0.017515	0.332659	0.13125
STMN 3	COPD&CP	6	3.023114	0.012136	0.330785	0.166667
NAP1L 3	COPD&CP	5	4.816302	0.009715	0.207628	0.2
EGFR		4	3.043796	0.020572	0.328537	0.261278
NRF1		4	3.50365	0.007751	0.285417	0.255981
HSPB 1		4	2.695864	0.028541	0.370939	0.273157
SQSTM 1		4	2.603406	0.029332	0.384112	0.273666
YWHA B		4	3.055961	0.022253	0.327229	0.259494

### Immune cell infiltration

**Fig. 5:**
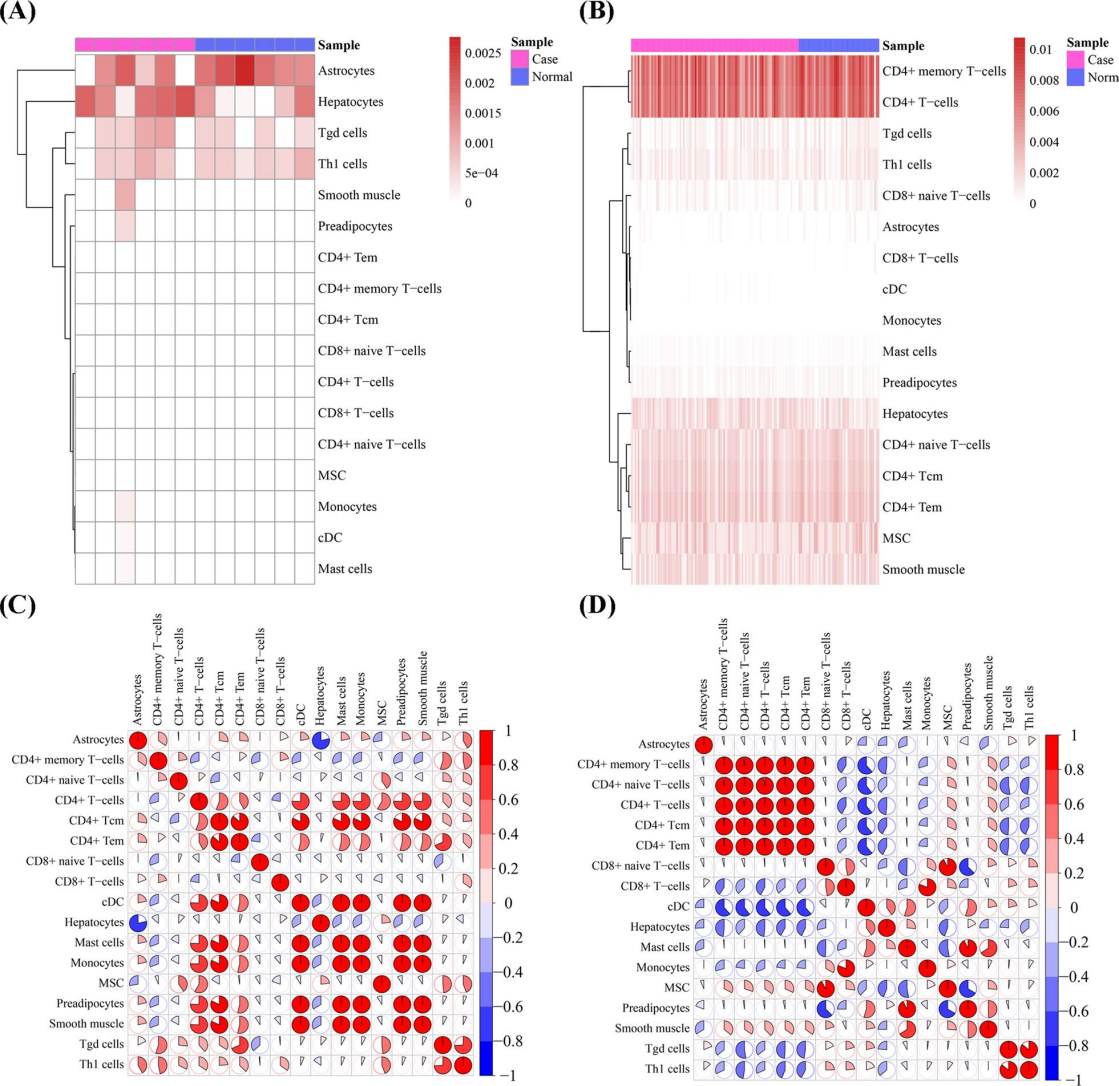
Cell type enrichment analysis in periodontitis (CP) and chronic obstructive pulmonary disease (COPD) for 22 potential crosstalk genes. (**A**) and (**B**) Heatmap of immune cell analysis for CP and COPD, respectively. The color legend represented the xCell scores. (**C**) and (**D**) the correlation among immune cells in CP and COPD, respectively. Both color and pie chart were corresponding to average Pearson coefficients

The infiltration score of 68 immune cells was analyzed with x Cell for the 22 potential crosstalk genes in CP and COPD. Because of a small number of genes in the current analysis, the input parameter of raw Enrichment Analysis [36] in x Cell packages [31] were adjusted. Thereby, the raw scores of immune cells for the 22 genes were acquired and corrected with transform Scores and spill Over method. Figure 5A, B show the plotted immune cell heat map, constructed with the pheatmap package of R project [37].

The closely related cell types of enriched immune cells based on the 22 crosstalk genes between CP and COPD are displayed in Fig. 5C, D. Thereby, Hepatocytes and Astrocytes were negatively correlated in CP (Fig. 5C). In COPD, CD4 + memory T-cells, CD4 + naive T-cells, CD4 + T-cells, CD4 + Tcm and CD4 + Tem were positively correlated to each other (Fig. 5D). Moreover, there were 8 cell pairs which were highly correlated to both CP and COPD (|correlation coefficients |> 0.5 and *p* < 0.05, Table 4).

**Table 4:** The highly correlated cell pairs in both CP and COPD

Cell	Cell	CP		COPD	
		cor	*p*Value	cor	*p*Value
CD4 + T-cells	cDC	0.755379	0.004497	− 0.61118	1.59E-16
CD4 + Tcm	CD4 + Tem	0.865414	0.000276	0.999716	7.8E-239
CD4 + Tcm	cDC	0.818692	0.001128	− 0.615	9.14E-17
cDC	Mast cells	1	4.25E-78	0.548103	5.58E-13
cDC	Preadipocytes	1	< 0.001	0.547586	5.93E-13
Mast cells	Preadipocytes	1	1.33E-79	0.925831	1.43E-63
Mast cells	Smooth muscle	1	< 0.001	0.659046	8.57E-20
Tgd cells	Th1 cells	0.743636	0.005564	0.855541	1.4E-43

**Fig. 6:**
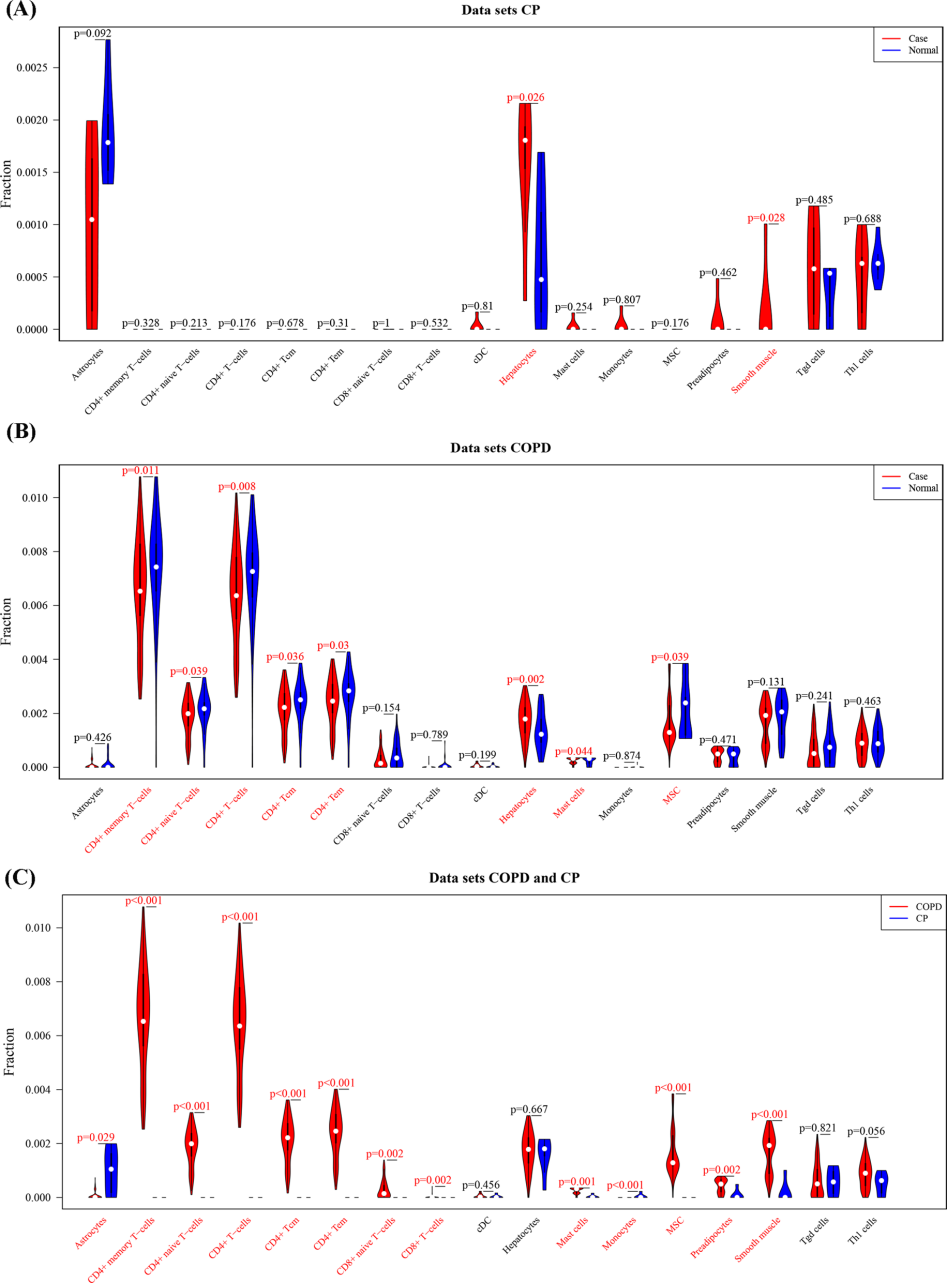
(**A**) and (**B**) different immune cell infiltration between disease and normal control samples for periodontitis (CP) and chronic obstructive pulmonary disease (COPD). **C** Different immune cell infiltration between CP disease and COPD disease samples

The immune cell infiltration difference of CP showed that Hepatocytes and Smooth muscle infiltrated more, compared with the control samples (Fig. 6A). For COPD, the results showed that Hepatocytes infiltrated more compared with the control samples, while CD4 + memory T-cells, CD4 + naive T-cells, CD4 + T-cells, CD4 + Tcm, CD4 + Tem and Mast cells infiltrated less (Fig. 6B). The results for COPD and CP showed that most immune cells were differently expressed between COPD and CP (Fig. 6C).

### Identification of crosstalk markers

**Fig. 7:**
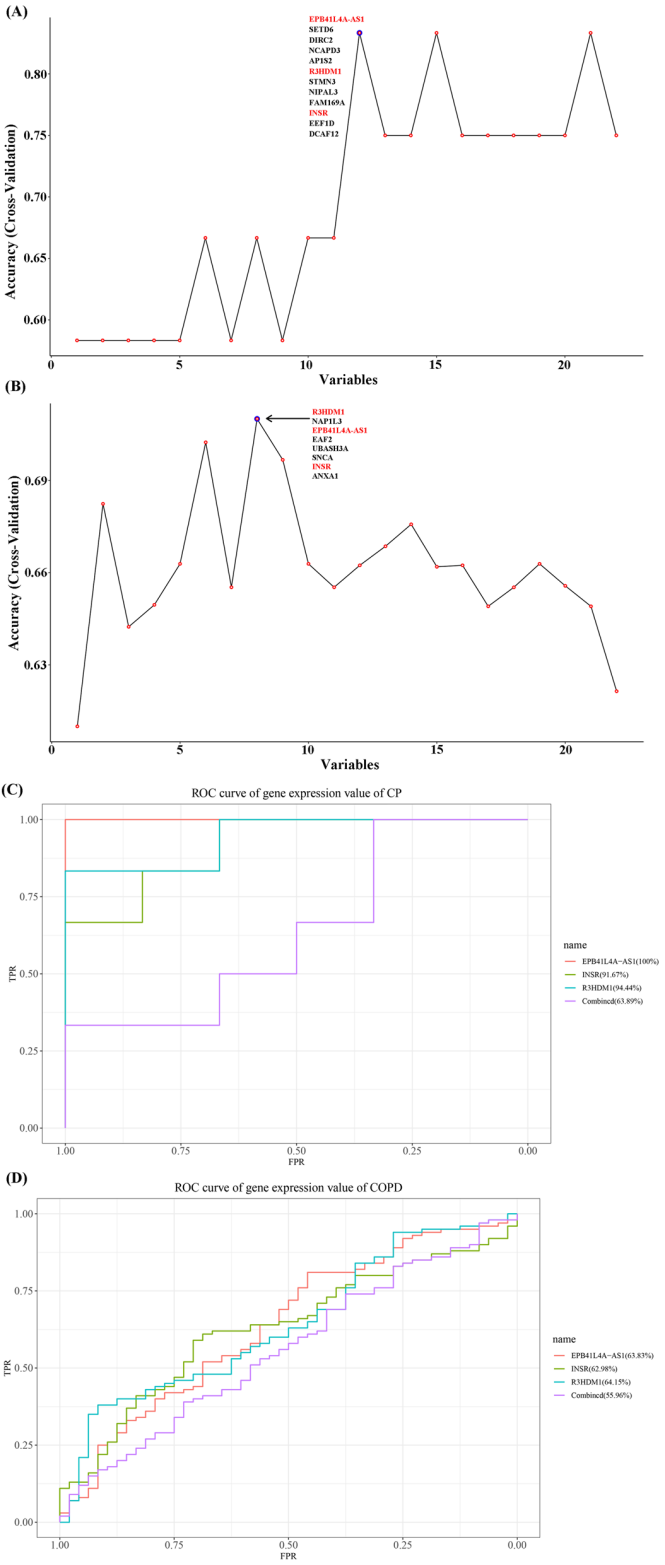
Feature genes selected by using RFE algorithm for periodontitis (CP) (**A**) and chronic obstructive pulmonary disease (COPD) (**B**). The abscissa of the figure is the variable of the number of genes, and the ordinate is the exact value of the whole data set measured under this variable. The results show that when the minimum variable is 6, the score is high, which means that 6 features could map the entire dataset. (**C**) and (**D**) the receiver operation curve (ROC) of 3 crosstalk marker genes and the combination for the 3 genes based on the average expression value in CP and COPD, respectively

**Fig. 8:**
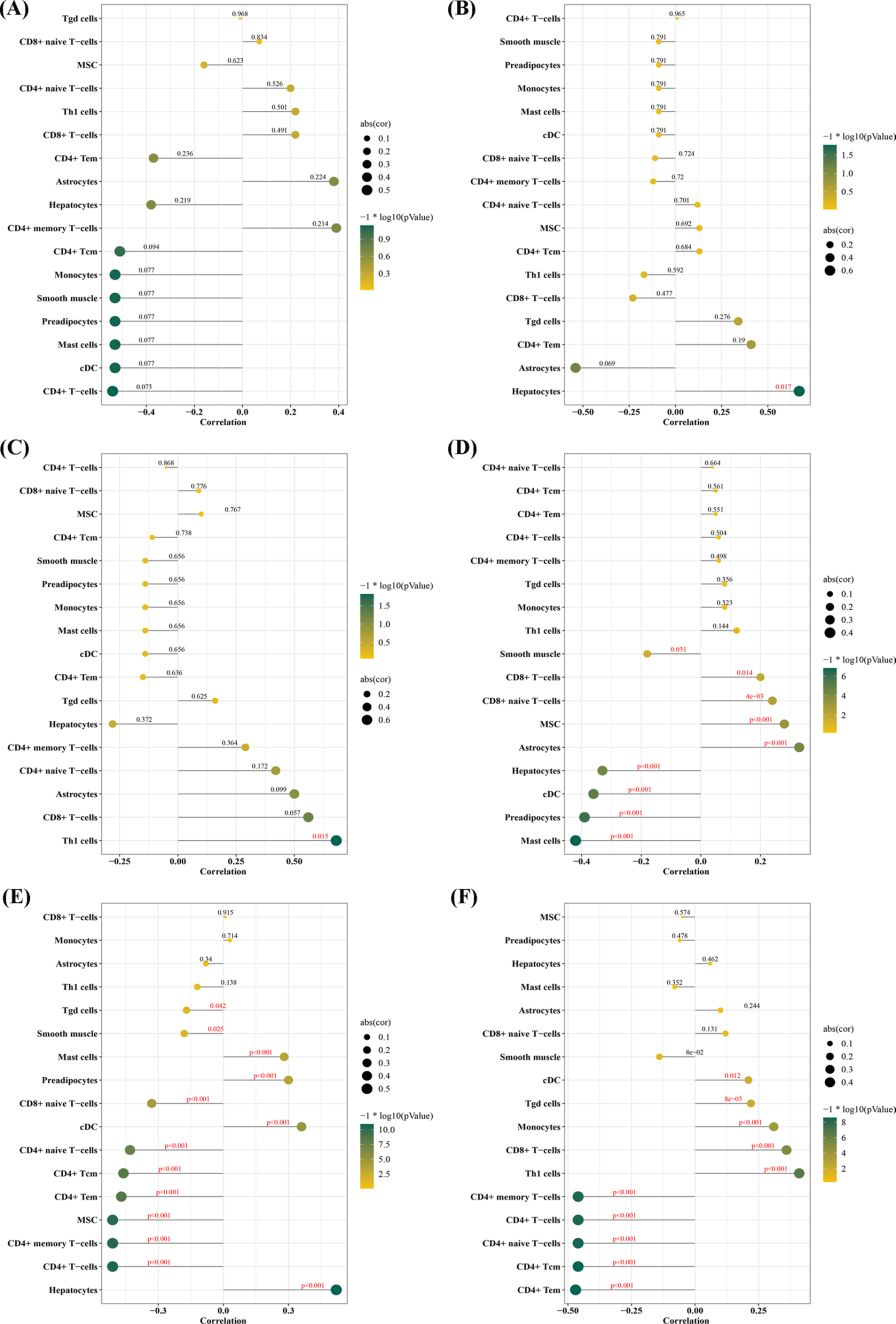
Correlation between EPB41L4A-AS1, INSR and R3HDM, and immune cells in periodontitis (CP) and chronic obstructive pulmonary disease (COPD). **A** Correlation between EPB41L4A-AS1 and infiltrating immune cells in CP. **B** Correlation between INSR and infiltrating immune cells in CP. **C** Correlation between R3HDM and infiltrating immune cells in CP. **D** Correlation between EPB41L4A-AS1 and infiltrating immune cells in COPD. **E** Correlation between INSR and infiltrating immune cells in COPD. **F** Correlation between R3HDM and infiltrating immune cells in COPD

By applying Recursive feature elimination (RFE) [32], 12 features for CP (Fig. 7A) and 8 features for COPD (Fig. 7B) were acquired, which had the highest prediction. Finally, the common features between CP and COPD were determined, whereby three genes were identified, i.e., EPB41L4A-AS1, INSR and R3HDM1. The prediction results, i.e., area under the curve (AUC) values for genes in CP and COPD are shown in Fig. 7C, D.

### Correlation analysis between crosstalk markers and infiltrating immune cells

Correlation analysis showed that EPB41L4A-AS1 was not correlated with any cells in CP (Fig. 8A). Furthermore, INSR was positively correlated with Hepatocytes in CP (r = 0.6714, *p* = 0.01679; Fig. 8B). R3HDM was positively correlated with Th1 cells in CP (r = 0.6783, *p* = 0.0153; Fig. 8C). In COPD, EPB41L4A-AS1 was positively correlated with Astrocytes (r = 0.330, *p* = 0.00004), and MSC (r = 0.2823, *p* = 0.0005) (Fig. 8D), and INSR was positively correlated with Hepatocytes in COPD (r = 0.5209, *p* < 0.001; Fig. 8E). Moreover, INSR was negatively correlated with CD4 + T-cells, CD4 + memory T-cells, MSC, CD4 + Tem, CD4 + Tcm, CD4 + naive T-cells, CD8 + naive T-cells, Smooth muscle and Tgd cells in COPD. R3HDM1 was negatively correlated with CD4 + Tem, CD4 + Tcm, CD4 + naive T-cells, CD4 + T-cells and CD4 + memory T-cells in COPD (Fig. 8F).

## Discussion

Main results: The current study revealed several potential crosstalk genes and correlated immune cell pairs between periodontitis and COPD. Three crosstalk genes were identified as the most relevant ones, i.e., EPB41L4A-AS1, INSR and R3HDM1. These three crosstalk markers correlated with different infiltrating immune cells. R3HDM was positively correlated with Th1 cells in both diseases, while INSR was positively correlated with Hepatocytes in periodontitis and COPD.

Comparison with literature and interpretation: This is the first bioinformatics analysis of the crosstalk and potential biological pathways between COPD and periodontitis. Accordingly, there is no directly comparable study available, which can be used for interpretation of the findings. Nevertheless, several results of the current study can support some pathophysiological links in the relationship between periodontitis and COPD, based on shared risk factors and inflammatory mechanisms. Considering the revealed biological processes and pathways (Fig. 3A, B), the presumption of shared risk factors appears supported; carbohydrate metabolism alongside with type II diabetes mellitus and the regulation of lipolysis in adipocytes can support the role of diabetes, obesity and the metabolic syndrome in the interplay between periodontitis and COPD. The available literature shows a clinical association between diabetes and periodontitis [38] as well as COPD [39], between obesity and periodontitis [40] as well as COPD [41] and same results for metabolic syndrome [9, 10]. Furthermore, the shared risk factor cigarette smoking [7, 8] can be related to several findings of the current analysis. In assessment of infiltrating immune cells, CD4 + and CD8 + cells were conspicuous (see Fig. 4). Cigarette smoking was reported to affect the innate and adaptive immunity, especially T helper cells, as well as CD4 + and CD8 + cells [42]. Accordingly, an influence of smoking on these cell types might lead to an autoimmunity resulting in pulmonal (COPD) and oral (periodontitis) inflammation. Therefore, the confounding effect of smoking appears highly relevant. This effect might rely on an affection of immune cells due to ingredients of cigarettes, resulting in an increased inflammatory burden, which can foster both COPD and periodontitis.

For a deeper comprehension of potentially related processes, the three crosstalk genes and related immune cells can be regarded. First, down-regulated EPB41L4A-AS1 was found as a potential crosstalk gene, of which no infiltrating immune cell was correlated to both, periodontitis and COPD. Regardless, this gene might play a potential role in the interrelation between the two diseases. Down regulation of EPB41L4A-AS1 was reported to activate nuclear factor kappa B (NF-κB) signaling pathway and to enhance inflammatory response in diabetes-related inflammation [43]. This might support an interaction via increase of systemic inflammation and could argue for the hypothesis of chronic systemic inflammatory syndrome [13]. Moreover, EPB41L4A-AS1 was identified as a potential biomarker for lung cancer [43]. Thereby, periodontitis [44] as well as COPD [45] potentially increase the risk of cancer, indicating EPB41L4A-AS1 as potentially relevant crosstalk marker in this context. INSR, i.e., insulin receptor was also a potential crosstalk gene. This gene has a high relevance in T cell immunity during inflammation [46]. A previous bioinformatics study revealed INSR to be regulated by miR-146a-5p in COPD patients [47]. This miRNA species has already been described to be of relevance in periodontal inflammation, especially with regard to lipopolysaccharide driven inflammation [48]. As immune cell, hepatocytes were found to be correlated to INSR in both periodontitis and COPD in the current study. On the one hand, *Prophyromonas gingivalis*, a gram-negative, anaerobic bacterium with high periodontal pathogenic potential, was found to affect hepatocytes [49, 50]. On the other hand, hepatocyte growth factor was increased in saliva of smokers with periodontitis [51]. Similarly, smokers suffering from COPD showed higher levels of hepatocyte growth factor in bronchial lavage [52]. Therefore, INSR and related hepatocytes and/or hepatocyte growth factors might be a shared genetic and immunological marker, especially in smokers affected by periodontitis and COPD. Lastly, R3HDM1 was found to be a potentially relevant crosstalk gene, which was especially correlated to Th1-cells in both diseases. This would underline a shared pathophysiology on immunological level and might be one key element to understand the relation between periodontitis and COPD. T helper cells, alongside with CD4 + and CD8 + T cells play a crucial role in the immunology of COPD [53]. Similarly, these cells are of importance in periodontal inflammation [54]. Furthermore, a recent review article highlighted the relevance of Th cell driven immunity in the pathogenesis of periodontitis and immune-mediated inflammatory diseases [55]. A recent animal study in mice showed that periodontitis affects course of Th1 profile cells and related cytokines (especially interferon gamma), leading to pulmonary alterations [56]. In this context, the importance of innate immunity, primarily of neutrophils as a key effector cell in inflammation and involved factor in the causal interrelationship between periodontitis and COPD can be highlighted [5]. Altogether, the T-cell mediated inflammation appears highly relevant in the pathophysiological processes of periodontitis and COPD. This again may support the hypothesis of a chronic systemic inflammatory syndrome [13]. Periodontitis and COPD may be an inflammatory disease of oxidative stress [57] and may be directly related as part of an underlying chronic inflammatory syndrome. This is supported by the strong relationship between those two diseases, which has been extensively studied in recent systematic reviews [58, 59]. Altogether, different hypotheses of interaction between COPD and periodontal diseases can be derived from the results: first, potentially periodontal pathogenic bacteria and their related virulence factors could trigger both, periodontal and pulmonal inflammation. Second, shared risk factors, especially smoking appear of relevance. Third, both diseases seem to be related to increase of T-cell mediated inflammation. Therefore, these might be the pathophysiological key points relating both conditions within one chronic systemic inflammatory syndrome. Thus, it appears to be an interesting hypothesis that COPD and periodontitis would be symptoms of an underlying inflammatory disease, rather than two singular entities. However, this hypothesis remains speculative and cannot be confirmed by the current analysis. In clinical consequence, both diseases need a shared understanding, therapy and prevention in dental and general practice. Thereby, the control (and elimination) of shared risk factors and reduction of inflammation are joint therapeutic measures. Potentially, more comprehensive therapeutic strategies are needed, including an interdisciplinary care approach for those diseases.

Strengths and limitations: This is the first bioinformatics study on periodontitis and COPD. The analysis was comprehensive and included evaluation of pathways, processes and infiltrating immune cells. The results are of potential clinical relevance, as they can serve as a theoretical basis for future studies in the field and might help to understand the shared pathophysiology of the two examined diseases. Nevertheless, several limitations must be addressed. Firstly, all the results are only on transcriptomic level, what is caused by the analysis applied in this current study. No clinical experiments were performed to confirm the bioinformatics results, making experimental validations of the findings as a subsequent study needed. The absence of a validation of the results either in-vitro or in a clinical setting makes all derived conclusions somewhat speculative; however, the hypothesis of a chronic systemic inflammatory syndrome is still a theoretical construct, where the role of periodontal diseases remains unclear. Thus, the findings of the current study open a new approach in the understanding of those two diseases. In addition to the missing validation, this study is limited by several other weakening points of a bioinformatics analysis, i.e., the inclusion of different patients with periodontitis and COPD, no consideration of patient specific data (e.g., age, gender, smoking habits, medication, co-morbidities) and thus a potentially very heterogeneous sample. Altogether, it must be stated that it is hardly possible to prove the shared mechanisms in a clinical setting, because each individual patient is unique and affected by a high variety of genetic, epigenetic, environmental and lifestyle factors. Therefore, although it appears a reasonable approach to foster a validation of the findings, it remains unclear if this will be unequivocally possible. As an additional limitation, the sample for periodontitis (CP) was quite small. This limits the ability to draw robust conclusions. Therefore, the findings must be seen as preliminary theoretical results.

## Conclusion

EPB41L4A-AS1, INSR and R3HDM1 are potential crosstalk genes between periodontitis and COPD. Especially Th1 cells and Hepatocytes might be relevant in the pathophysiological relationship between the two diseases. It might be conceivable that periodontitis and COPD are related within a chronic systemic inflammatory syndrome. These findings can serve as a basis for future studies and should be evaluated in experimental and/or clinical investigations.

## Data Availability

The data analyzed in the current study are available in the GEO database. The original contributions presented in the study are included in the article; further inquiries can be directed to the corresponding author.

## Abbreviations

AUC: Area under curve
COPD: Chronic obstructive pulmonary disease
CP: Chronic periodontitis
DEG: Differentially expressed gene
GEO: Gene expression omnibus
PPI: Protein–protein interaction
RFE: Recursive feature elimination
ROC: Receiver operation characteristic
